# Trends of Prevalence Estimates and Risk Factors of Depressive Symptoms among Healthcare Workers Over one Year of the COVID-19 Pandemic

**DOI:** 10.2174/17450179-v18-e2206160

**Published:** 2022-06-30

**Authors:** Ahmed Yassin, Abdel-Hameed Al-Mistarehi, Ola Soudah, Reema Karasneh, Sayer Al-Azzam, Aref A. Qarqash, Aws G. Khasawneh, Kefah A. Alhayk, Majdi Al Qawasmeh, Raid M. Kofahi, Salma Y. Bashayreh, Khalid El-Salem

**Affiliations:** 1 Department of Neurology, Faculty of Medicine, Jordan University of Science and Technology, Irbid, Jordan; 2 Department of Public Health and Family Medicine, Faculty of Medicine, Jordan University of Science and Technology, Irbid, Jordan; 3 Department of Basic Medical Sciences, Faculty of Medicine, Yarmouk University, Irbid, Jordan; 4 Department of Clinical Pharmacy, Jordan University of Science and Technology, Irbid, Jordan; 5 Faculty of Medicine, Jordan University of Science and Technology, Irbid, Jordan; 6 Department of Psychiatry, Faculty of Medicine, Jordan University of Science and Technology, Irbid, Jordan

**Keywords:** COVID-19, Depression, Healthcare workers, Health providers, Physicians, One-year

## Abstract

**Background:**

COVID-19 pandemic has an overwhelming psychologic burden on healthcare workers (HCWs). This study aims to investigate the changes in the prevalence, estimates, severity, and risk factors of depressive symptoms among HCWs within the first year of the COVID-19 pandemic.

**Methods:**

An observational e-survey collected data on HCWs’ socio-demographic characteristics, occupational situation, and depressive symptoms as measured by Patient Health Questionnaire–9 (PHQ-9). The e-survey was distributed one month after the onset of the COVID-19 pandemic (onset group) and again after one year (one-year group).

**Results:**

A total of 422 HCWs were included (Mean (SD) age, 35.3 (9.9) years; 71.3% males), with 211 (50%) participants in each group. In the total cohort, the mean PHQ-9 score was 8.5, and 36.7% reported clinically significant levels of depressive symptoms with a PHQ-9 score of ≥10. Compared to the onset group, the one-year group reported a higher risk of major depressive disorder (41.7% *vs*. 31.8%; OR 1.538; 95%CI 1.032–2.291; *p=0.034*), a higher mean PHQ-9 score (9.5 (6.8) *vs*. 7.4 (5.3), *p<0.001*), and more severe depressive symptoms *(p<0.005*). Participants who were younger, unmarried, underwent testing for COVID-19, reported lower monthly income, did not receive special COVID-19 education, or had lower satisfaction with institutional preparedness had significantly higher depression scores and symptoms in both onset and one-year groups (*p<0.05* for each category). Female gender and direct contact with COVID-19 patients or samples were significant risk factors within the onset group. Occupation as a physician, history of COVID-19 testing or infection, and perception of significant changes in work schedule or intensity were significantly associated with higher depression scores and symptoms among the one-year group.

**Conclusion:**

This study sheds light on an unspoken but significant rise in prevalence estimates and severity of depressive symptoms among HCWs over a year of the COVID-19 pandemic and shows the vulnerable subgroups for whom a psychological intervention might be warranted.

## 1 INTRODUCTION

Coronavirus Disease 2019 (COVID-19) pandemic and its associated precautionary measures have affected life aspects across the globe in all possible ways [1-10]. According to the Johns Hopkins Coronavirus Resource Center, by the end of January 2022, more than 200 nations have been affected by the Severe Acute Respiratory Syndrome Coronavirus 2 (SARS-CoV-2), the cause of COVID-19, with more than 370 million confirmed cases and over 5.6 million deaths globally [11]. Although COVID-19 presents mainly with respiratory manifestations, its complications are vast and could include cardiovascular, thromboembolic, and neurological ones [12-16]. Besides, COVID-19 harms the psychosocial well-being of patients and the general public as a whole [17-19]. A Swedish cross-sectional study conducted early in the Pandemic from March 26 to April 5, 2020, found that 22.2% of the general population reported clinically significant levels of depressive symptoms (PHQ-9 ≥ 10) [18].

Healthcare workers (HCWs), who are on the frontline, have also been significantly affected. A study with a total of 1978 participants in the general population across the United States of America (USA) found that employment as an HCW was a significant risk factor for depression [20]. The negative effect of the pandemic on HCWs likely results from their perceived risk of acquiring the infection upon direct contact with suspected or confirmed cases [21]. Other contributing factors include the increase in the HCWs’ workload, their worries about transmitting the infection to other patients, loved ones, colleagues, and family members, their negative feelings that progressively build up as they see the patients’ suffering and anxiety about their families, the periods of lockdown and movement restriction, and the adverse effects of the pandemic on their social support system [21-24]. Thus, HCWs are vulnerable to emotional distress and psychological challenges, resulting in stress, depression, and anxiety symptoms [25-31].

**Fig. (1):**
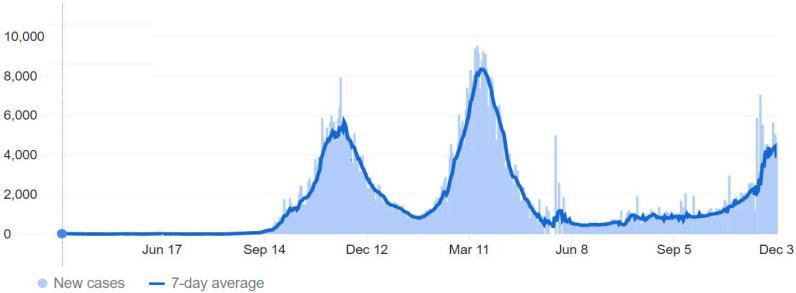
Coronavirus Disease 2019 (COVID-19) daily and weekly average new cases in Jordan over 2020-2021. The first case was confirmed on March 02, 2020; the first peak was around November 2020, and the second one was around April 2020. The figure was reprinted and adapted with permission from COVID-19 Dashboard by the Center for Systems Science and Engineering (CSSE) at Johns Hopkins University (JHU) [11, 34].

On March 2, 2020, the first COVID-19 case was confirmed in Jordan, with no new reported cases of COVID-19 until March 15, when 11 new cases had been tested positive for COVID-19, followed by a rise in cases in the following days and weeks [13, 32]. Accordingly, on March 17, the government enforced different levels of lockdown for three months. Thus, the number of cases was relatively low throughout this period. A few months after the lockdown’s end, around October-November 2020, the number of cases started to rise significantly until we reached the first peak. Consequently, partial restrictions on people’s movement were imposed, including a curfew after 9 pm on weekdays and a complete lockdown on weekends. Numbers got relatively under control towards the end of 2020. However, later in February and March 2021, cases started to rise again significantly (second peak), resulting in more tightening of the restrictions [11, 33, 34] (Fig. **1**). This COVID-19 should be a reminder to be prepared for the potential public health and psychiatric challenges of emerging pandemics in the future [35].

To our knowledge, this study is one of the first to assess the trend in prevalence rates, severity degree, and risk factors for symptoms of depression among HCWs over the first year of the COVID-19 pandemic. The study achieves this goal by investigating depressive symptoms among HCWs approximately one month after the onset of the COVID-19 pandemic (onset group) and again after one year during the second peak (one-year group). The ultimate goal is to recommend interventions to alleviate depressive symptoms, particularly for vulnerable subgroups, and potentially prevent these symptoms from occurring if similar health crises occur in the future. Such recommendations would be directed to health care providers' and administrators' attention at institutional, national, and even international levels.

## 2 MATERIALS AND METHODS

### 2.1 Study Design, Population, and Ethical Approval

This study is an exploratory observational survey conducted online using the Google Form tool. To achieve the study objectives, the e-survey was conducted *via* two-stage sampling. The e-survey was firstly distributed after about one month of COVID-19 pandemic onset in Jordan, between the 15^th^ and 30^th^ of April 2020. Then, the same questionnaire was distributed again after one year of COVID-19 pandemic onset between the 15^th^ and 30^th^ of March 2021. Thus, the total cohort consisted of two subgroups: onset and one-year groups. Participants were eligible if they were HCWs, 18 years of age or older, living in Jordan, and had internet access *via* smartphone or computer. The study investigators shared the e-survey link *via* social media platforms, mainly WhatsApp, and asked the participants to further disseminate the e-survey to their peers. On receiving and clicking the link, participants would be auto-directed to the informed consent page, and a short message describing the objectives and design of the study followed by a consent question would appear. If they agree to participate, they would be further directed to the survey questions; unless the form will terminate. Participants could terminate the survey at any time desired. The survey was anonymous, and information confidentiality was assured. Participants did not receive any compensation or rewards for their participation in the study.

The study protocol was revised and ethically approved by the Institutional Review Board (IRB) of the research and ethics committee at Jordan University of Science and Technology, Irbid, Jordan (IRB number 106/132/2020). This study was conducted following the 1975 Helsinki Declaration, revised in 2008, and later amendments or comparable ethical standards. This work has been reported based on the STROBE statement guidelines for reporting observational studies [36].

### 2.2 Survey Instruments

The participants self-reported the data, and the questionnaire was designed based on the literature [18, 20, 37-42]. The questionnaire validity was checked by a pilot study that included 20 random HCWs who assessed the questionnaire’s clarity, and no significant modifications were required. The questionnaire consisted of 3 sections, including socio-demographic characteristics, occupational situation, and depressive symptoms.

Participants first completed socio-demographic characteristics, including age, gender, area of living, marital status, and whether they were living with an elderly of 65 years or older (yes/no). Then, they were asked about their personal history of COVID-19 testing, infection, and the need for hospital admission if they got infected. The history of getting the anti-COVID-19 vaccine was investigated within the one-year sample, as the vaccine was unavailable at the onset of the pandemic [43].

The occupational situation was assessed using questions asked about the participant’s working position, monthly income in Jordanian Dinar (JD), whether they were in direct contact with confirmed or suspected COVID-19 individuals or samples during the work (yes/no), the estimated number of confirmed or suspected COVID-19 individuals or samples that participants dealt with, and whether they received an exceptional education to deal with COVID-19 patients (yes/no). Participants’ perception of contact with COVID-19 patients was also assessed using a 5-point Likert scale, ranging from “1=low level of contact” to “5=high level of contact”. Furthermore, participants’ evaluation of their institution's preparedness to deal with COVID-19 patients was studied using a 6-point Likert scale ranging from “Very bad” to “Excellent”. In addition, the perceived level of change in work schedule and intensity due to the COVID-19 pandemic was assessed with response options of no perceived changes / A little / some / much / very much.

The third part of the questionnaire assessed depression symptoms experienced by the participants using the Patient Health Questionnaire–9 (PHQ-9), a validated and reliable clinical and research tool for major depressive disorder (MDD) screening and depression severity grading [44-46]. PHQ-9 consists of nine items asking about nine symptoms based on the diagnostic criteria for MDD in the Diagnostic and Statistical Manual of Mental Disorders, Fourth Edition (DSM-IV). The frequency of each symptom was assessed using a 4-point Likert scale (0 = not at all, 1 = several days, 2 = more than half of the days, 3 = nearly every day), then a total score ranging from 0 to 27 was obtained by summing the items scores. Depressive symptoms were described in three formats; PHQ-9 scores, severity categories of depression, and binary categorization into high risk and low risk for MDD determined by a total score cutoff of 10, which was found to have a sensitivity of 88% and a specificity of 85-88% for predicting MDD [44-46]. Severity categories were defined as normal (score 0-4), mild (score 5-9), moderate (score 10-14), moderately severe (score 15-19), and severe (score, 20-27) [44]. Among our participants, the Cronbach’s alfa (α) for the items on the PHQ-9 depression scale was 0.903.

### 2.3 Statistical Analysis

All data analyses were performed using the IBM Statistical Package for the Social Sciences (SPSS) software for Windows, version 25.0. Continuous variables, including age, perceived level of contact with COVID-19 patients, and PHQ-9 scale total scores, were presented as mean ± standard deviation (m±SD) after verifying the normality of the dataset. The age variable was further presented as a categorical variable with four groups, based on the interquartile range, as 23-27, 28-31, 32-39, and ≥40 years. Descriptive statistics were conducted to calculate the frequencies and percentages for the categorical variables. Internal consistency reliability was measured using Cronbach’s α for the PHQ-9 scale.

The differences between onset and one-year samples were analyzed using a chi-square test for categorical variables, including socio-demographics, occupational characteristics, and severity categories of depression. In contrast, Student’s t-test or one-way ANOVA was used for continuous variables, including PHQ-9 total scores and perceived level of contact with COVID-19 patients. Moreover, we investigated the differences in the PHQ-9 scale total scores among each sample using Student’s t-test or one-way ANOVA. The differences in the severity categories of depression among each sample were assessed using the chi-square test.

Binary logistic regression analyses were used to estimate the Odds Ratio (OR) and 95% Confidence Interval (95% CI) for MDD risk factors among each sample of HCWs. Model selection using the stepwise backward approach with a cutoff *p-value of 0.2* was used to select the final, most parsimonious model where age, gender, marriage status, living with the elderly, occupation, monthly income, COVID-19 vaccination, previous testing, previous infection, direct contact with COVID-19 patients or samples during the work, getting an exceptional education to deal with COVID-19 patients, participant’s evaluation of institutional preparedness, and perceived changes in work schedule due to COVID-19 pandemic were included as independent explanatory variables. The variables in the last model were checked for multicollinearity using the variance inflation factor (VIF). Statistical significance was considered at a *p-value of ≤ 0.05*.

## 3 RESULTS

### 3.1 Participants’ Characteristics

In this study, from 494 HCWs invited to participate (239 onset and 253 one-year groups), 427 respondents initiated the survey with a participation rate of 86.4%. Of the respondents, 422 (98.8%) completed the survey items and were included in the final sample (Figs. **2A** and **2B**).

### 3.2 Total Cohort Characteristics

The participants' age ranged from 23 to 73 years with a mean (SD) of 35.3 (9.9) years, and 71.3% were males. Of the total cohort, 254 (60.2%) were married, and 168 (39.8%) were single, widowed, or divorced. Most participants (n=344, 81.5%) were physicians, while 78 (18.5%) were nurses, pharmacists, or technicians. More than half of the participants (58.1%) reported a low monthly income (less than one thousand Jordanian Dinar-JD).

### 3.3 The Onset and One-year Sample Characteristics

Each group of the two samples included 211 participants, representing 50% of the total cohort. A similar age mean (SD) were observed in the two samples (24-70 years, 34.7 (9.3) in the onset sample, and 23-73 years, 35.8 (10.5) in the one-year sample). Among the onset group, 73.0% were males, 62.6% were married, 77.7% were physicians, and 62.5% reported a low monthly income. Among the one-year sample, 69.7% were males, 57.8% were married, 85.3% were physicians, and 53.6% informed a low monthly income. The two groups matched age, gender, marital status, occupation, and monthly income (*p>0.05*). Table **1** shows the participants' characteristics in the total cohort and the onset and one-year groups.

In the one-year group, the proportions of HCWs who got tested for (87.2%) or infected with (46.0%) COVID-19 were significantly higher than that of the onset group (23.2%, 0.5%, respectively) (*p<0.001* for each).

### 3.4 Occupational Situation

HCWs’ contact with COVID-19 patients and samples in the one-year group was significantly higher than the rate in the onset group (*p<0.001*). There were no significant differences between the two groups in the percentage of HCWs who received special education to deal with COVID-19 patients. Compared to the onset sample, more participants in the one-year sample reported poor evaluation of institutional preparedness to deal with COVID-19 patients. There were no significant differences in the perception of changes in work schedule and intensity between the two groups (*p=0.474*).

#### 3.4.1 Trends of Depressive Symptoms among HCWs over a year of the COVID-19 Pandemic

In the total cohort, 155 HCWs (36.7%) reported clinically significant levels of depressive symptoms, indicating a high risk for MDD. Their mean (SD) PHQ-9 score was 8.5 (6.2), and the severity of depression symptoms was mild in 33.4%, moderate in 20.4%, moderately severe in 9.5%, and severe in 6.9%.

**Fig. (3):**
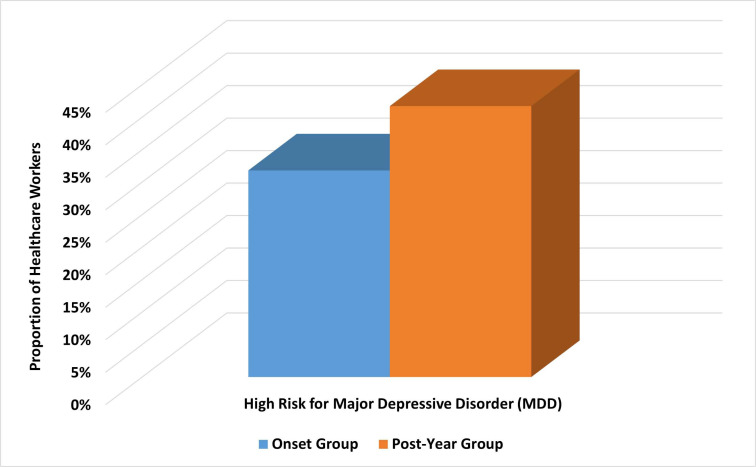
Trends of prevalence estimates of major depressive disorder (MDD) among health care workers within a year of the COVID-19 pandemic.

For the one-year group, 88 participants (41.7%) had a high risk for MDD compared to 67 participants (31.8%) in the onset group (Fig. **3**). This difference was statistically significant with an unadjusted OR of 1.538; 95% CI, 1.032–2.291; *p=0.034*. Moreover, the mean (SD) score of the PHQ-9 scale for depression was significantly higher in the one-year sample compared with the onset sample (9.5 (6.8) *vs*. 7.4 (5.3), *p<0.001*). Moreover, the one-year group reported more severe depression symptoms than the onset group *(p=0.005*). Table **2** shows the scores and severity of depression symptoms among HCWs in the total cohort and onset and one-year groups.

#### 3.4.2 Factors Associated with Depressive Symptoms in the Onset Group

In the onset sample, younger participants, women, and unmarried participants had significantly higher depression scores than their counterparts Table **3**. Data from this sample showed a trend of significantly decreasing depression scores with increasing monthly income. Moreover, HCWs who had undergone testing for COVID-19 reported higher mean scores on PHQ-9 than those who had not (10.35 (6.34) *vs*. 6.54 (4.63), *p<0.001)*. Similarly, higher depression mean scores were observed among HCWs with direct contact with COVID-19 patients and samples than those who did not report such contact (10.42 (6.29) *vs*. 6.45 (4.56), *p<0.001*). HCWs who did not receive a special COVID-19 education had significantly higher scores on the PHQ-9 scale than those who did (8.05 (5.69) *vs*. 5.83 (3.73), *p=0.006*). Lastly, poor evaluation of institutional COVID-19 preparedness was significantly associated with higher depression scores Table **3**.

Regarding the severity categories of depression, similar to the previous findings based on PHQ-9 scores, HCWs who were younger, women, unmarried, or had a lower monthly income experienced significantly more severe symptoms of depression than their counterparts. Moreover, HCWs who underwent testing for SARS-CoV-2 infection, had direct contact with COVID-19 patients or samples, did not receive special COVID-19 education, and were unsatisfied with the institution's preparedness had more severe depressive symptoms than their counterparts Table **3**.

Living with the elderly, occupation, and perceived changes in work schedule or intensity were not associated with depression (score or severity categories) in the onset group (*p>0.05*).

#### 3.4.3 Factors Associated with Depressive Symptoms in the One-year Group

One-year participants had higher scores and severity levels of depression symptoms than the onset group. However, the factors associated with significantly higher depression scores and more severe symptoms were to a large degree similar to those observed in the onset group. One-year participants who were younger, women, unmarried, had lower monthly income, had direct contact with COVID-19 patients or samples, did not receive special COVID-19 education, or were unsatisfied with institutional preparedness had higher depression scores and severity compared to their counterparts, Table **4**.

However, a few risk factors were different between the onset and the one-year groups. Physicians in the one-year group had higher mean scores of depression (9.53 (6.75)) than in the onset group (7.43 (5.31)) with a mean difference of 2.10 (t(420) = 3.56, *p<0.001*) for PHQ-9 depression score. Moreover, unlike the onset group, physicians in the one-year group experienced significantly more severe depressive symptoms than other HCWs (*p=0.009*). Unlike the onset group participants, data from the one-year sample indicated that perceived more work schedule and intensity changes were associated with more depression symptoms (*p<0.001*). Among the one-year group, approximately half of the participants (46.0%) reported a history of COVID-19 infection, and they had significantly higher PHQ-9 scores and depression severity levels than those who did not get infected (*p<0.05*).

Among the one-year group, about two-thirds (71.6%) of HCWs became vaccinated against COVID-19 one year after the pandemic onset, and the depression mean (SD) score was lower among vaccinated participants (8.90 (6.13)) than those who did not get the vaccine (9.78 (6.99)). However, this difference was not statistically significant, with a *p-value of 0.394*. Living with the elderly was insignificant for depression scores and severity among the onset and one-year samples (*p>0.05*).

#### 3.4.4 Risk Factors of Major Depressive Disorder (MDD) among HCWs

Binary logistic regression analyses showed that, after controlling for confounders, low monthly income, lack of special education to deal with COVID-19 patients, and poor evaluation of institutional preparedness were independent risk factors for developing MDD among both onset and one-year samples (Table **5**). Female gender (OR, 2.31; 95% C.I. 1.13–4.74; *p=0.022*) and having direct contact with COVID-19 patients or samples (OR, 2.27; 95% C.I. 1.01–5.09; *p=0.046*) were significant risk factors for developing clinically depressive symptoms among the onset sample only. In contrast, undergoing a test for COVID-19 was an independent risk factor for developing MDD symptoms in the one-year sample only (OR, 4.48; 95% C.I. 1.07–18.81; *p=0.041*).

## 4 DISCUSSION

To our knowledge, this study is one of the first to investigate the change in prevalence rates and risk factors of depressive symptoms among HCWs over a year during the COVID-19 pandemic. Our study showed a high proportion of HCWs manifesting depressive symptoms with a significant increase in the prevalence rate and severity of symptoms over the first year of the pandemic in Jordan (32% at onset *vs*. 42% after one year). This study necessitates the importance of applying mental health support interventions for HCWs during pandemics, including but not limited to communication support, appropriate work shifts, and good communication between the team leader and staff [47]. The change in factors associated with depressive symptoms was not impressive. HCWs who were younger, unmarried, did not receive special COVID-19 education, had lower satisfaction with institutional preparedness, and had lower monthly income were at higher risk for depression and had more severe symptoms in both onset and one-year groups. However, unlike the one-year group, female gender and direct contact with COVID-19 patients or samples were significant risk factors within the onset group. On the other hand, occupation (physician), perceived changes in work schedule or intensity, and being tested for COVID-19 were significantly associated with depressive symptoms within the one-year group. As expected, the one-year group had way higher rates of COVID-19 infection with significantly higher depression scores and severity. The anti-COVID19 vaccine was received by two-thirds of the one-year group and was considered a relative, but insignificant, protective action.

### 4.1 Depression among HCWs

Previous studies showed a significant but unspoken high depression risk among HCWs during the COVID-19 pandemic, similar to our study [37, 40, 48, 49]. A multicentric survey-based study in China using the HPQ-9 questionnaire with 1257 HCWs found that 50% manifested symptoms of depression [37]. A study in northeast Italy found that 26.6% of HCWs had at least moderate depression [40]. Another study in southeast Ireland reported positive depression scores among 42.6% of HCWs working in acute hospital settings [48]. A multicenter, cross-sectional study on HCWs in Ghana conducted from July 11 to August 12, 2020, found that 21.1% of participants had depression [49]. However, none of these studies trended depression scores and severity among HCWs over a longer duration as our study investigated.

Other studies on the general public compared depression scores before and during the pandemic [39, 50]. A study that looked at the change in the prevalence estimates of depressive symptoms among US adults before and during COVID-19 showed a 3-fold increase in depression prevalence estimates during the pandemic [39]. A Chinese study found higher depression scores among people in 2020, particularly those living in more heavily impacted provinces than among the 2016 sample [50]. Another Chinese study based on the Chinese undergraduate cohort showed that new Chinese undergraduate students had no worsening of depressive symptoms after three months of mass quarantine for COVID-19 [51]. However, all these studies trended depressive symptoms in the general population rather than HCWs as we did in our study.

#### 4.1.1 Factors Associated with Depressive Symptoms

##### 4.1.1.1 Shared Depression-associated Factors between Onset and One-year Groups

Our study showed that younger age and unmarried status were persistent risk factors for depression, regardless of time. Similar to our findings, previous studies have shown that younger adults had more depressed moods during the COVID-19 pandemic [18, 50, 52]. The movement restrictions and social isolation associated with the pandemic could have resulted in more depressive symptoms [53, 54]. Such social isolation could negatively impact younger unmarried HCWs as they are usually more socially active than older married HCWs. Besides, the literature reported that marriage and the presence of supportive social systems as protective factors against depression during the COVID-19 pandemic [18, 39]. French and Spanish studies showed that feelings of loneliness increased the risk of depression during COVID-19 lockdown [3, 52]. A recent study from Denmark compared the risk of stress, depression, and functional impairment before lockdown, before March 11, 2020, with that risk during the lockdown and among re-interviewees in July 2021 [55]. The authors found fewer depressive symptoms among adults with children living at home.

There was no significant difference in the percentages of HCWs who received special education to deal with COVID-19 between the onset and one-year groups. Lack of such education was a risk factor for depression, using binary logistic regression, in both groups of similar significance. This finding indicates that lacking knowledge about a new health crisis has a persistent similar negative impact on the psychology of HCWs over time.

Even though one-year participants were less satisfied with the institutional preparedness to deal with COVID-19 patients than the onset group, which is likely an expected result of the increased burden of the pandemic over time, low satisfaction was a significant risk factor for depression, using binary logistic regression, in both groups.

Our study found that lower monthly income is a significant risk factor for depression, using binary logistic regression, in both onset and one-year groups. This observation indicates that limited income is a constant risk factor for depression regardless of the timing of a health crisis. Many studies showed a similar negative impact of low economic status, financial problems, and losing a job on the psychological immunity of adults to stressful times during COVID-19 [3, 18, 39, 47, 50, 56]. Financial support, on the other hand, such as the government's tax-free salary relief, was shown to counteract these adverse psychological effects [49].

##### 4.1.1.2 Depression-associated Factors at the Onset of the Pandemic

Unlike the one-year group, the female gender was a significant risk factor for MDD, using binary logistic regression in the onset group. This finding is concordant with previous studies’ results which indicated that females in general and female HCWs had a higher risk and more severe degree of depression than males [3, 17, 30, 37, 40-42, 48, 50, 57]. This could be attributed to the fact that women usually tend to be more worried and careful and display higher levels of fear of COVID-19 about themselves and their families at the beginning of the non-previously experienced and unpredicted stressful event [56]. Moreover, some mood changes and depressed feelings could be attributed to hormonal changes in women [58, 59].

Our findings showed that direct contact with COVID-19 patients or samples was a risk factor for depression, using binary logistic regression, in the onset group only. Previous studies showed that HCWs directly engaged with COVID-19 patients had a higher risk and more severe degrees of depression [3, 37, 40, 41, 50, 57]. However, direct contact with COVID19 patients was significantly lower in the onset group than in the one-year group. This might reflect the amplified perception of risk for acquiring and transmitting infection on contact with COVID-19 patients and samples at the beginning of the pandemic, which eased up over time and with growing experience in dealing with such patients/samples. W. Lu *et al*. (2020) reported that frontline medical workers with close contact with infected COVID-19 patients have higher fear, anxiety, and depression scores than administrative staff [25].

##### 4.1.1.3 Depression-associated Factors after One Year of the Pandemic

Our study showed that among HCWs, physicians had significantly more depressive symptoms within the one-year group. This finding indicated that physicians likely had the highest cumulative psychological burden over a year of work among HCWs, given their direct responsibility for managing patients with COVID-19 and dealing with their morbidity and mortality with the increased number of cases [60-62]. Thus, the increasing trend of depression symptoms among our cohort after one year of the pandemic onset could also be attributed to the higher practical burden, increased responsibilities of providing families with regular updates, and HCWs may feel more socially isolated, more concerned for their health, and helpless for not having helped or supported the patient enough with high COVID-19 rates in light of reported lack sufficient knowledge about data sharing and patient confidentiality [62, 63]. A cross-sectional, web-based study investigating the mental health outcomes among HCWs during the COVID-19 pandemic in Italy found that general practitioners were more likely to endorse posttraumatic stress disorder (PTSD) than other HCWs [64]. Moreover, a recent systematic review reported higher prevalence rates of depression among physicians (40.4%) than nurses (28%) [61].

There were no significant differences in the perception of changes in work schedule and intensity between the two groups. However, the change in work schedule and intensity resulted in higher depressive symptoms over time. This finding emphasizes the importance of stress chronicity as a risk factor for developing depressive symptoms. A study from the USA on the general population found that the prevalence of depression during the pandemic was statistically significantly higher among those who worked full time compared with part-timers and unemployed people [20].

The testing for COVID-19 was a risk factor, using binary logistic regression, for developing depressive symptoms in the one-year sample. More frequent testing for COVID-19 could result in frequent and more prolonged contact with patients with confirmed or suspected infection, which per se increases stress over time and results in depressive symptoms. Vaccination became routine across HCWs after one year of the pandemic, and although not statistically significant, COVID-19 vaccination was associated with lower depression mean (SD) scores among vaccinated participants than unvaccinated ones. This finding is concordant with a previous study that reported an association between getting the first dose of the anti-COVID-19 vaccine and significant improvement in mental health [65].

### 4.2 Study Limitations

This study has a few limitations. It is a single-center study, the sample size of participants was relatively small, and the achieved representativeness was low; thus, limiting our findings to other populations. Thus, the results are unlikely to be generalizable beyond the people who responded. However, the novelty of this study is in its timeliness as it investigates the changes in depressive symptoms over a year of the COVID-19 pandemic. The study method of using an observational internet-based survey with its inherent limitations, such as recall bias and the lack of available data on non-respondents, are other limitations that could affect the results’ interpretation. A selection bias cannot be ruled out as the participants needed access to a smartphone/computer to participate, limiting our sample's generalizability. However, the internet-based survey is a cost-effective approach for data collection that provides a safe and private environment for the participants to give accurate and honest information. The study did not survey the same HCWs to see the actual trend in their depression scores, severity, and risk factors. However, this limitation was compensated for by the fact that the onset and one-year groups matched in age, gender, marital status, occupation, and monthly income. Most respondents were physicians and males, which, although relatively similar in both onset and one-year surveys, makes the generalizability of this study results to all HCWs and particularly female HCWs less accurate. However, this can partly be explained by the fact that most of the HCWs in Jordan (70%) are males [66]. Also, this study did not investigate the coping mechanisms for depression among HCWs and the possible psychiatric and cognitive effects of physical activity, diet, sleep habits, smoking status, nicotine dependence, comorbidities, disabilities, and laboratory investigations [17, 38, 67-74]. Thus, future studies with a larger cohort of HCWs examined a wide range of potential factors for depression and investigated possible coping skills and interventions are suggested.

## CONCLUSION

Health care providers have a high prevalence estimate of depressive symptoms, which has increased, along with depression severity, over the first year of the COVID-19 pandemic. Factors associated with depression have not changed significantly over time. Persistently vulnerable HCWs included those who were young, unmarried, did not receive special COVID-19 education, had lower satisfaction with institutional preparedness, or had lower monthly income. Female gender and direct contact with COVID-19 patients or samples were significant risk factors for pandemic onset. While physicians, HCWs with intense work schedules, those who underwent a test for COVID-19, and those who got infected with COVID-19 had higher rates and more severe depressive symptoms one year after the pandemic onset. We emphasize the urgent need for health care officials to implement exceptional interventions, strategies, and policies to promote mental health wellness among HCWs during the COVID-19 pandemic.

## Figures and Tables

**Fig. (2A):**
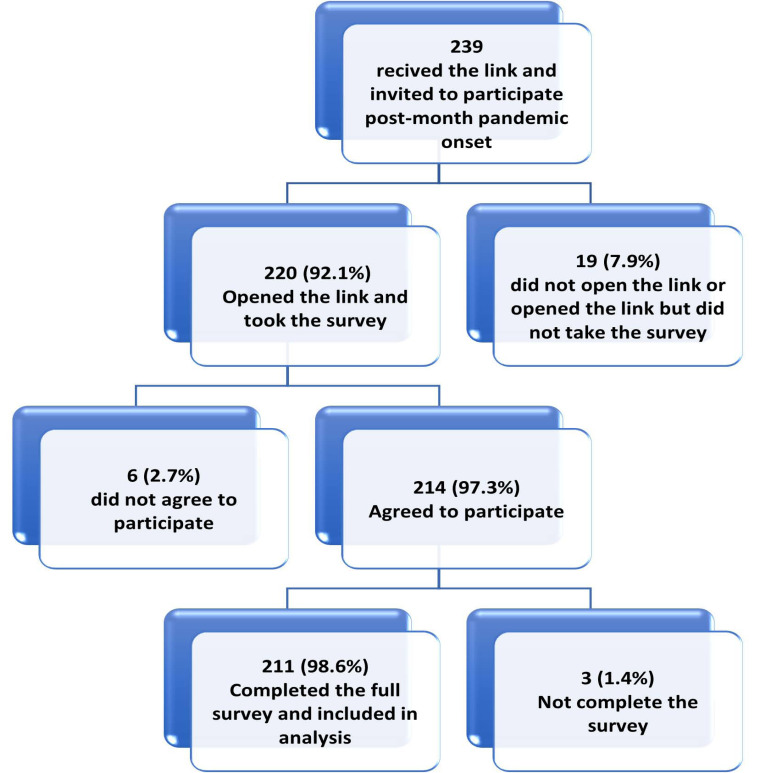
Study participants' flow chart one month after COVID-19 pandemic onset.

**Fig. (2B):**
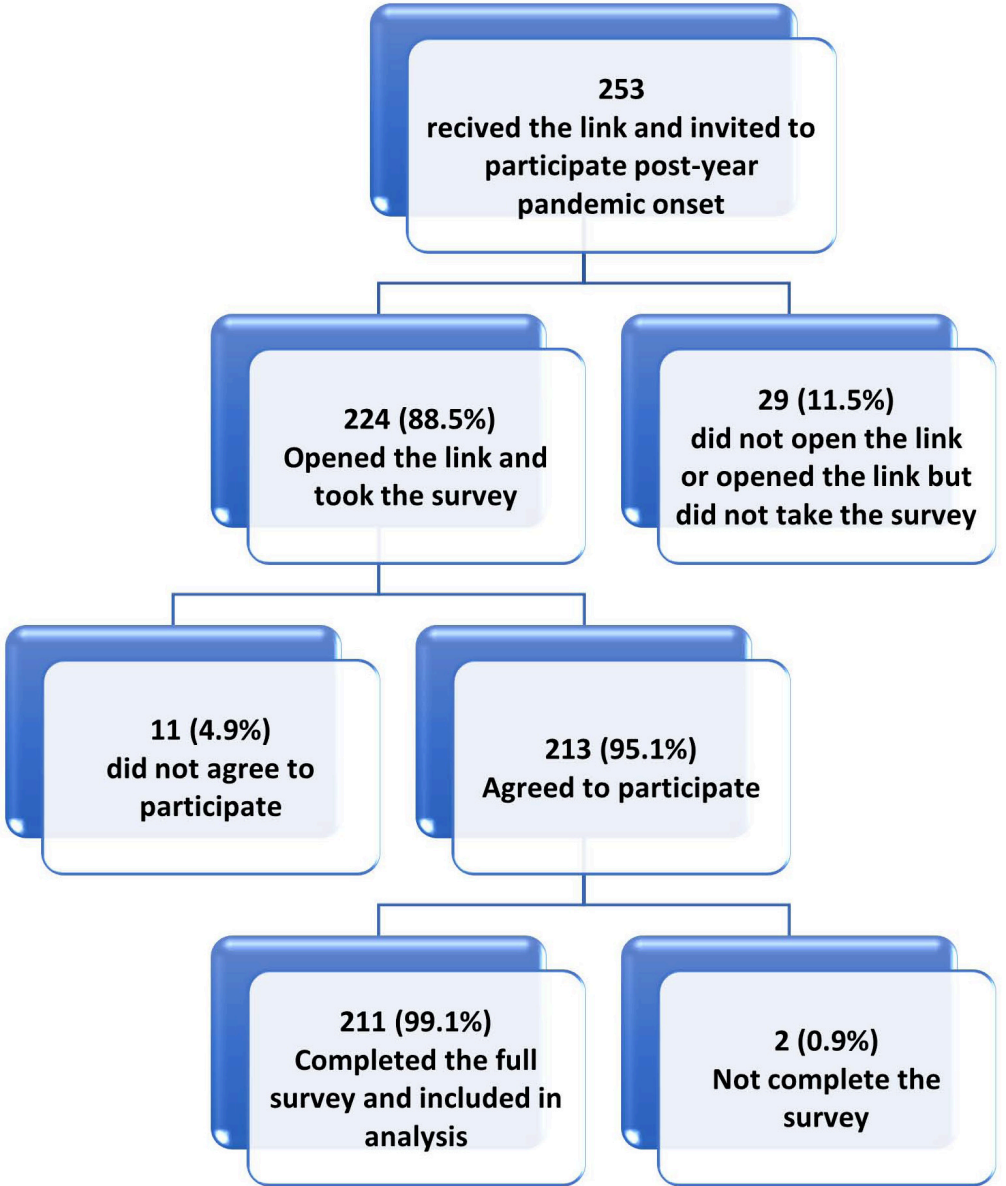
Study participants' flow chart one year after COVID-19 pandemic onset.

**Table 1:** Socio-demographic and occupational characteristics of the survey participants in total cohort and subgroups.

**Characteristic**	**Total Cohort, n=422** **n (%)**	**Onset Group, n=211** **n (%)**	**One-year Group, n=211** **n (%)**	** p-value **
** Age, y ® **				
23-27	90 (21.3)	47 (22.3)	43 (20.4)	0.761
28-31	107 (25.4)	56 (26.5)	51 (24.2)	
32-39	118 (28.0)	59 (28.0)	59 (28.0)	
≥40	107 (25.4)	49 (23.2)	58 (27.5)	
** Gender **				
Male	301 (71.3)	154 (73.0)	147 (69.7)	0.451
Female	121 (28.7)	57 (27.0)	64 (30.3)	
** Marital status **				
Unmarried*	168 (39.8)	79 (37.4)	89 (42.2)	0.320
Married	254 (60.2)	132 (62.6)	122 (57.8)	
** Living with elderly of 65 years old or older **				
No	217 (51.4)	125 (59.2)	92 (43.6)	0.001
Yes	205 (48.6)	86 (40.8)	119 (56.4)	
** Occupation **				
Physician	344 (81.5)	164 (77.7)	180 (85.3)	0.060
Others†	78 (18.5)	47 (22.3)	31 (14.7)	
** Monthly income, Jordanian Dinar (JD) **				
<500	56 (13.3)	25 (11.8)	31 (14.7)	0.066
500-1000	189 (44.8)	107 (50.7)	82 (38.9)	
1000-2000	66 (15.6)	33 (15.6)	33 (15.6)	
>2000	111 (26.3)	46 (21.8)	65 (30.8)	
** COVID-19 characteristics **				
Vaccinated against COVID-19^¥^	-	-	151 (71.6)	-
Tested for COVID-19	233 (55.2)	49 (23.2)	184 (87.2)	<0.001
Hx of COVID-19 infection	98 (23.2)	1 (0.5)	97 (46.0)	<0.001
Hx of hospital admission due to COVID-19 infection (% out of infected persons)	5 (5.1)	0 (0.0)	5 (5.2)	-
Direct contact with confirmed or suspected COVID-19 individuals or samples	197 (46.7)	52 (24.6)	145 (68.7)	<0.001
Perceived level of contact with COVID-19 patients, Mean (SD) (score range; 1-5)	3.09 (1.30)	2.59 (1.19)	3.59 (1.20)	<0.001
** Estimated number of confirmed or suspected COVID-19 cases that participants dealt with **				
Zero	227 (53.8)	160 (75.8)	67 (31.8)	<0.001
1-49	91 (21.6)	31 (14.7)	60 (28.4)	
50-100	40 (9.5)	9 (4.3)	31 (14.7)	
>100	64 (15.2)	11 (5.2)	53 (25.1)	
** Receiving an exceptional education to deal with COVID-19 patients **				
No	293 (69.4)	152 (72.0)	141 (66.8)	0.245
Yes	129 (30.6)	59 (28.0)	70 (33.2)	
** Participants’ evaluations of their institution preparedness to deal with COVID-19 patients **				
Very bad	26 (6.2)	9 (4.3)	17 (8.1)	0.002
Bad	63 (14.9)	25 (11.8)	38 (18.0)	
Fair	98 (23.2)	38 (18.0)	60 (28.4)	
Good	116 (27.5)	65 (30.8)	51 (24.2)	
Very good	92 (21.8)	57 (27.0)	35 (16.6)	
Excellent	27 (6.4)	17 (8.1)	10 (4.7)	
** Perceived changes in work schedule and intensity due to COVID-19 pandemic **				
No perceived changes	27 (6.4)	10 (4.7)	17 (8.1)	0.474
A little	31 (7.3)	19 (9.0)	12 (5.7)	
Some	78 (18.5)	39 (18.5)	39 (18.5)	
Much	165 (39.1)	81 (38.4)	84 (39.8)	
Very much	121 (28.7)	62 (29.4)	59 (28.0)	

The Chi-square test assessed the differences between onset and one-year samples for socio-demographic and occupational characteristics. The student’s t-test was used to estimate the difference in means of perceived level of contact with COVID-19 patients between the two groups.

® Age was defined as a categorical variable with four groups, divided approximately at the interquartile ranges.

*Unmarried category included single (never married), widowed, and divorced participants.

† Others included nurses, pharmacists, and technicians.

¥ COVID-19 vaccine was not available at the onset of the COVID-19 pandemic.

**Table 2:** Scores and severity categories of depression in total cohort and subgroups.

**Characteristic**	**Total Cohort, n=422**	**Onset Group, n=211**	**One-year Group, n=211**	***p-value***
***PHQ-9, depression symptoms***				
Total score, Mean (SD)	8.48 (6.16)	7.43 (5.31)	9.53 (6.75)	*<0.001*
**Depression Severity Categories, n (%)**				
Normal	126 (29.9)	69 (32.7)	57 (27.0)	*0.005*
Mild	141 (33.4)	75 (35.5)	66 (31.3)	
Moderate	86 (20.4)	47 (22.3)	39 (18.5)	
Moderately severe	40 (9.5)	12 (5.7)	28 (13.3)	
Severe	29 (6.9)	8 (3.8)	21 (10.0)	

One-way ANOVA was used to estimate the difference in means of PHQ-9 scores, while the chi-square test was conducted to assess the differences in depression severity categories.

Abbreviations: PHQ-9, 9-item Patient Health Questionnaire

**Table 3:** Differences in the scores and severity categories of depression among the onset group (n=211).

**Characteristic**	** PHQ-9, Depression Symptoms **						
	**Total Score, Mean (SD)**	** p-value **	** Depression Severity Categories, n (%) **				
			**Normal**	**Mild**	**Moderate**	**Moderately Severe & Severe**	** p-value **
** Age, y **							
23-27	9.53 (5.70)	<0.001	10 (21.3)	12 (25.5)	18 (38.3)	7 (14.9)	0.002
28-31	7.61 (5.41)		18 (32.1)	20 (35.7)	12 (21.4)	6 (10.7)	
32-39	7.81 (4.99)		15 (25.4)	26 (44.1)	11 (18.6)	7 (11.9)	
≥40	4.73 (4.08)		26 (53.1)	17 (34.7)	6 (12.2)	0 (0.0)	
** Gender **							
Male	6.55 (5.18)	<0.001	63 (40.9)	53 (34.4)	25 (16.2)	13 (8.4)	<0.001
Female	9.79 (4.95)		6 (10.5)	22 (38.6)	22 (38.6)	7 (12.3)	
** Marriage status **							
Unmarried*	9.46 (5.95)	<0.001	17 (21.5)	24 (30.4)	24 (30.4)	14 (17.7)	<0.001
Married	6.21 (4.49)		52 (39.4)	51 (38.6)	23 (17.4)	6 (4.5)	
** Living with elderly of 65 years old or older **							
No	7.25 (5.67)	0.557	46 (36.8)	41 (32.8)	24 (19.2)	14 (11.2)	0.214
Yes	7.69 (4.76)		23 (26.7)	34 (39.5)	23 (26.7)	6 (7.0)	
** Occupation **							
Physician	7.65 (5.56)	0.249	54 (32.9)	54 (32.9)	39 (23.8)	17 (10.4)	0.425
Others^†^	6.64 (4.29)		15 (31.9)	21 (44.7)	8 (17.0)	3 (6.4)	
** Monthly income, Jordanian Dinar (JD) **							
<500	9.76 (6.09)	<0.001	4 (16.0)	12 (48.0)	4 (16.0)	5 (20.0)	<0.001
500-1000	8.97 (4.96)		19 (17.8)	41 (38.3)	33 (30.8)	14 (13.1)	
1000-2000	4.33 (3.96)		20 (60.6)	9 (27.3)	4 (12.1)	0 (0.0)	
>2000	4.78 (4.45)		26 (56.5)	13 (28.3)	6 (13.0)	1 (2.2)	
** COVID-19 characteristics **							
COVID-19 tested	10.35 (6.34)	<0.001	11 (22.4)	13 (26.5)	14 (28.6)	11 (22.4)	0.001
Direct contact with COVID-19 pts. or samples	10.42 (6.29)	<0.001	11 (21.2)	13 (25.0)	15 (28.8)	13 (25.0)	<0.001
Perceived contact with COVID-19 pts, Mean (SD)	-	-	2.23 (1.19)	2.65 (1.11)	2.70 (1.18)	3.30 (1.17)	0.002
** Estimated number of confirmed or suspected COVID-19 that participants dealt with **							
Zero	6.33 (4.26)	<0.001	56 (35.0)	67 (41.9)	33 (20.6)	4 (2.5)	<0.001
1-49	8.19 (5.95)		13 (41.9)	6 (19.4)	6 (19.4)	6 (19.4)	
50-100	13.56 (5.59)		0 (0.0)	2 (22.2)	4 (44.4)	3 (33.3)	
>100	16.27 (5.57)		0 (0.0)	0 (0.0)	4 (36.4)	7 (63.6)	
** Receiving an exceptional education to deal with COVID-19 patients **							
No	8.05 (5.69)	0.006	46 (30.3)	49 (32.2)	39 (25.7)	18 (11.8)	0.034
Yes	5.83 (3.73)		23 (39.0)	26 (44.1)	8 (13.6)	2 (3.4)	
** Participants’ evaluations of their institution preparedness to deal with COVID-19 patients **							
Very bad	11.11 (5.51)	<0.001	0 (0.0)	3 (33.3)	5 (55.6)	1 (11.1)	<0.001
Bad	10.80 (5.79)		4 (16.0)	6 (24.0)	9 (36.0)	6 (24.0)	
Fair	8.87 (5.63)		8 (21.1)	13 (34.2)	12 (31.6)	5 (13.2)	
Good	7.20 (4.76)		18 (27.7)	28 (43.1)	14 (21.5)	5 (7.7)	
Very good	5.46 (4.44)		28 (49.1)	21 (36.8)	6 (10.5)	2 (3.5)	
Excellent	4.76 (4.45)		11 (64.7)	4 (23.5)	1 (5.9)	1 (5.9)	
** Perceived changes in work schedule and intensity due to COVID-19 pandemic **							
No changes	6.40 (3.81)	0.247	4 (40.0)	4 (40.0)	2 (20.0)	0 (0.0)	0.305
A little	5.74 (5.11)		7 (36.8)	10 (52.6)	0 (0.0)	2 (10.5)	
Some	6.77 (5.94)		16 (41.0)	13 (33.3)	6 (15,4)	4 (10.3)	
Much	7.44 (4.99)		26 (32.1)	29 (35.8)	18 (22.2)	8 (9.9)	
Very much	8.50 (5.48)		16 (25.8)	19 (30.6)	21 (33.9)	6 (9.7)	

Student’s t-test or one-way ANOVA was conducted to investigate the differences in the PHQ-9 scale scores with socio-demographic and occupational characteristics. In contrast, the differences in the severity categories of depression were assessed using a chi-square test.

*Unmarried category included single, widowed, and divorced participants.

† Others included nurses, pharmacists, and technicians.

**Table 4:** Differences in the scores and severity categories of depression among the one-year group (n=211).

**Characteristic**	** PHQ-9, Depression Symptoms **						
	**Total Score, Mean (SD)**	** p-value **	** Depression Severity Categories, n (%) **				
			**Normal**	**Mild**	**Moderate**	**Moderately Severe & Severe**	** p-value **
** Age, y **							
23-27	11.79 (6.71)	<0.001	6 (14.0)	13 (30.2)	7 (16.3)	17 (39.5)	0.001
28-31	11.71 (6.83)		6 (11.8)	19 (37.3)	11 (21.6)	15 (29.4)	
32-39	9.02 (6.18)		19 (32.2)	16 (27.1)	15 (25.4)	9 (15.3)	
≥40	6.47 (6.09)		26 (44.8)	18 (31.0)	6 (10.3)	8 (13.8)	
** Gender **							
Male	8.48 (6.68)	0.001	50 (34.0)	45 (30.6)	24 (16.3)	28 (19.0)	0.003
Female	11.94 (6.35)		7 (10.9)	21 (32.8)	15 (23.4)	21 (32.8)	
** Marriage status **							
Unmarried^*^	11.69 (6.41)	<0.001	13 (14.6)	29 (32.6)	15 (16.9)	32 (36.0)	<0.001
Married	7.96 (6.59)		44 (36.1)	37 (30.3)	24 (19.7)	17 (13.9)	
** Living with elderly of 65 years old or older **							
No	9.24 (6.87)	0.582	27 (29.3)	29 (31.5)	17 (18.5)	19 (20.7)	0.850
Yes	9.76 (6.68)		30 (25.2)	37 (31.1)	22 (18.5)	30 (25.2)	
** Occupation **							
Physician	10.06 (6.95)	0.006	45 (25.0)	55 (30.6)	31 (17.2)	49 (27.2)	0.009
Others^†^	6.48 (4.49)		13 (41.9)	13 (41.9)	4 (12.9)	1 (3.2)	
** Monthly income, Jordanian Dinar (JD) **							
<500	16.87 (4.71)	<0.001	1 (3.2)	1 (3.2)	6 (19.4)	23 (74.2)	<0.001
500-1000	10.28 (6.21)		13 (15.9)	31 (37.8)	23 (28.0)	15 (18.3)	
1000-2000	7.33 (6.22)		14 (42.4)	12 (36.4)	2 (6.1)	5 (15.2)	
>2000	6.20 (5.53)		29 (44.6)	22 (33.8)	8 (12.3)	6 (9.2)	
** COVID-19 characteristics **							
Vaccinated	8.90 (6.13)	0.394	37 (24.5)	53 (35.1)	21 (13.9)	40 (26.5)	0.007
COVID-19 tested	10.10 (6.73)	0.001	43 (23.4)	56 (30.4)	39 (21.2)	46 (25.0)	0.002
COVID-19 infected	10.71 (6.64)	0.019	20 (20.6)	27 (27.8)	22 (22.7)	28 (28.9)	0.046
Direct contact with COVID-19 pts. or samples	10.85 (6.85)	<0.001	29 (20.0)	45 (31.0)	28 (19.3)	43 (29.7)	0.001
Perceived contact with COVID-19 pts, Mean (SD)	-	-	3.02 (1.33)	3.48 (1.04)	3.72 (1.21)	4.29 (0.87)	<0.001
** Estimated number of confirmed or suspected COVID-19 that participants were dealt with **							
Zero	6.66 (5.54)	<0.001	28 (41.8)	19 (28.4)	15 (22.4)	5 (7.5)	<0.001
1-49	8.68 (5.74)		15 (25.0)	27 (45.0)	9 (15.0)	9 (15.0)	
50-100	11.19 (7.17)		9 (29.0)	3 (9.7)	6 (19.4)	13 (41.9)	
>100	13.15 (7.19)		5 (9.4)	17 (32.1)	9 (17.0)	22 (41.5)	
** Receiving an exceptional education to deal with COVID-19 patients **							
No	10.80 (7.05)	<0.001	28 (19.9)	44 (31.2)	25 (17.7)	44 (31.2)	<0.001
Yes	6.97 (5.29)		29 (41.4)	22 (31.4)	14 (20.0)	5 (7.1)	
** Participants’ evaluations of their institution preparedness to deal with COVID-19 patients **							
Very bad	17.88 (5.10)	<0.001	0 (0.0)	1 (5.9)	4 (23.5)	12 (70.6)	<0.001
Bad	10.92 (6.99)		9 (23.7)	9 (23.7)	7 (18.4)	13 (34.2)	
Fair	9.55 (5.25)		11 (18.3)	22 (36.7)	16 (26.7)	11 (18.3)	
Good	7.71 (6.66)		18 (35.3)	19 (37.3)	6 (11.8)	8 (15.7)	
Very good	7.09 (6.37)		14 (40.0)	12 (34.3)	5 (14.3)	4 (11.4)	
Excellent	7.80 (7.21)		5 (50.0)	3 (30.0)	1 (10.0)	1 (10.0)	
** Perceived changes in work schedule and intensity due to COVID-19 pandemic **							
No changes	8.82 (6.25)	<0.001	8 (47.1)	1 (5.9)	5 (29.4)	3 (17.6)	<0.001
A little	7.92 (6.78)		3 (25.0)	6 (50.0)	2 (16.7)	1 (8.3)	
Some	7.03 (4.99)		15 (38.5)	15 (38.5)	5 (12.8)	4 (10.3)	
Much	8.81 (6.66)		25 (29.8)	30 (35.7)	9 (10.7)	20 (23.8)	
Very much	12.75 (7.06)		6 (10.2)	14 (23.7)	18 (30.5)	21 (35.6)	

Student’s t-test or one-way ANOVA was conducted to investigate the differences in the PHQ-9 scale scores with socio-demographic and occupational characteristics. In contrast, the differences in the severity categories of depression were assessed using a chi-square test.

*Unmarried category included single, widowed, and divorced participants.

† Others included nurses, pharmacists, and technicians.

**Table 5:** Risk factors for Major Depressive Disorder (MDD) among healthcare workers identified by binary logistic regression analyses*.

**Variable**	**No. of Disease Cases/** **No. of Total Cases (%)**	**Adjusted OR**	**95% CI**	** p-value **
** Onset sample (n=211) **				
**Gender**				
Male	38/154 (24.7)	REF	REF	REF
Female	29/57 (50.9)	2.312	1.127 – 4.744	** 0.022 **
**Monthly income, Jordanian Dinar (JD)**				
<500	9/25 (36.0)	1.077	0.281 – 4.126	0.914
500-1000	47/107 (43.9)	2.883	1.122 – 7.403	** 0.028 **
1000-2000	4/33 (12.1)	0.964	0.245 – 3.791	0.958
>2000	7/46 (15.2)	REF	REF	REF
**COVID-19 tested**				
Yes	25/49 (51.0)	2.258	0.951 – 5.358	0.065
No	42/162 (25.9)	REF	REF	REF
**Direct contact with COVID-19 pts. or samples**				
Yes	28/52 (53.8)	2.271	1.013 – 5.092	** 0.046 **
No	39/159 (24.5)	REF	REF	REF
**Receiving an exceptional education to deal with COVID-19 patients**				
Yes	10/59 (16.9)	REF	REF	REF
No	57/152 (37.5)	4.081	1.714 – 9.720	** 0.001 **
**Participants’ evaluations of institution preparedness to deal with COVID-19 patients**				
Very bad	6/9 (66.7)	6.155	1.119 – 19.089	** 0.034 **
Bad	15/25 (60.0)	6.603	1.173 – 37.160	** 0.032 **
Fair	17/38 (44.7)	3.987	0.768 – 20.687	0.100
Good	19/65 (29.2)	2.064	0.414 – 10.276	0.376
Very good	8/57 (14.0)	1.088	0.205 – 5.768	0.921
Excellent	2/17 (31.8)	REF	REF	REF
** One-year sample (n=211) **				
**Monthly income, Jordanian Dinar (JD)**				
<500	29/31 (93.5)	32.549	6.602 – 160.470	** <0.001 **
500-1000	38/82 (46.3)	2.296	1.043 – 5.058	** 0.039 **
1000-2000	7/33 (21.2)	0.687	0.234 – 2.018	0.495
>2000	14/65 (21.5)	REF	REF	REF
**COVID-19 tested**				
Yes	85/184 (46.2)	4.475	1.065 – 18.813	** 0.041 **
No	3/27 (11.1)	REF	REF	REF
**Direct contact with COVID-19 pts. or samples**				
Yes	71/145 (49.0)	1.932	0.882 – 4.232	0.099
**Bold indicates statistical significance with a p-value <0.05.**				
No	17/66 (25.8)	REF	REF	REF
**Receiving an exceptional education to deal with COVID-19 patients**				
Yes	19/70 (27.1)	REF	REF	REF
No	69/141 (48.9)	2.762	1.345 – 5.672	** 0.006 **
**Participants’ evaluations of institution preparedness to deal with COVID-19 patients**				
Very bad	16/17 (94.1)	21.338	1.423 – 320.057	** 0.027 **
Bad	20/38 (52.6)	2.096	0.343 – 12.817	0.423
Fair	27/60 (45.0)	1.645	0.282 – 9.591	0.580
Good	14/51 (27.5)	0.891	0.148 – 5.351	0.900
Very good	9/35 (25.7)	1.369	0.216 – 8.701	0.739
Excellent	2/10 (20.0)	REF	REF	REF

* Socio-demographic characteristics (including age, gender, marriage status, living with elderly, occupation, and monthly income), COVID-19 characteristics (including vaccination status, previous testing, previous infection, and direct contact with COVID-19 patients or samples), getting an exceptional education to deal with COVID-19 patients, participants’ evaluations of institution preparedness, and perceived changes in work schedule and intensity due to COVID-19 pandemic were included as independent explanatory variables in the backward stepwise binary logistic regression model.

## Data Availability

The datasets generated and analyzed during the current study are available from the corresponding author [A.Y].
